# Cardiovascular disease risk perception among community adults in South China: a latent profile analysis

**DOI:** 10.3389/fpubh.2023.1073121

**Published:** 2023-05-09

**Authors:** Zhiting Guo, Yong Yuan, Yujia Fu, Nianqi Cui, Qunfei Yu, Erling Guo, Chuanqi Ding, Yuping Zhang, Jingfen Jin

**Affiliations:** ^1^Nursing Department, The Second Affiliated Hospital of Zhejiang University School of Medicine, Hangzhou, Zhejiang, China; ^2^Faculty of Nursing, Zhejiang University School of Medicine, Hangzhou, Zhejiang, China; ^3^China Mobile (Hangzhou) Information Technology Co., Ltd., Hangzhou, Zhejiang, China; ^4^School of Public Health, Hebei Medical University, Shijiangzhuang, Hebei, China; ^5^Emergency Department, Changxing County People's Hospital, Huzhou, Zhejiang, China; ^6^Key Laboratory of the Diagnosis and Treatment of Severe Trauma and Burn of Zhejiang Province, Hangzhou, Zhejiang, China

**Keywords:** cardiovascular disease, risk perception, knowledge, public education and awareness, latent profile analysis

## Abstract

**Objective:**

Risk perception, a critical psychological construct, influences health behavior modification and maintenance of individuals with cardiovascular disease (CVD) risk. Little is known about CVD risk perception among Chinese adults. This research examined the profiles of CVD risk perception of community adults in South China, and explored the characteristics and factors that influence their perception of CVD risk.

**Method:**

This cross-sectional study was conducted in Hangzhou, Zhejiang Province, in South China from March to July 2022 and included 692 participants. Risk perception was assessed using the Chinese version of the Attitude and Beliefs about Cardiovascular Disease Risk Questionnaire. Latent profile analysis (LPA) was performed to extract latent classes of CVD risk perception. These classes of CVD risk perception were compared with 10-year CVD risk categories to define correctness of estimation. Chi-square tests and multinomial regression analyses were used to identify differences between these categories.

**Results:**

Three CVD risk perception classes were identified by LPA: low risk perception (14.2% of participants), moderate risk perception (46.8%), high risk perception (39.0%). Individuals who were aged with 40–60 year (*OR* = 6.94, 95% *CI* = 1.86–25.84), diabetes (*OR* = 6.26, 95% *CI* = 1.34–29.17), married (*OR* = 4.52, 95% *CI* = 2.30–8.90), better subjective health status (*OR* = 3.23, 95% *CI* = 1.15–9.10) and perceived benefits and intention to change physical activity (*OR* = 1.16, 95% *CI* = 1.05–1.27) were more likely to be in the high-risk perception class. Compared to absolute 10-year CVD risk based on China-PAR, a third of participants (30.1%) correctly estimated their CVD risk, 63.3% overestimated it and 6.6% underestimated it. CVD risk underestimation was associated with hypertension (*OR* = 3.91, 95% *CI* = 1.79–8.54), drinking (*OR* = 3.05, 95% *CI* = 1.22–7.64), better subjective health status (*OR* = 2.67, 95% *CI* = 1.18–6.03).

**Conclusions:**

Most adults in South China possess a moderate level of CVD risk perception. Advanced age, higher monthly income, diabetes and better health status were significantly related to higher perceived CVD risk. Individuals with hypertension, drinking and better subjective health status were associated with CVD risk underestimation. Healthcare professionals should pay attention to the indicators for different classes and identify underestimation group as early as possible.

## Introduction

Cardiovascular disease (CVD) is a major cause of premature mortality and disease burden globally (1). As of 2019, CVD accounts for 46.74% and 44.26% of the causes of death in rural and urban areas in China, respectively, i.e., over two of every five deaths were due to CVD (2). The burden of CVD will continue to increase as China faces the dual pressures of an aging population and the widespread prevalence of metabolic risk factors (2). Risk factor control through evidence-based drug therapy and healthy behaviors are cost-effective public health interventions for reducing CVD risk by up to 80% (3). Nevertheless, recommended CVD preventive medication use and risk factor control have been suboptimal worldwide (4). According to the report of NCD risk factor collaboration, less than half hypertension patients were treated (47% for female, 38% for male) globally, and the control rate was 23% for female, 18% for male (5). In China, the proportion of primary drug use is low in high CVD risk individuals, with the use of blood pressure-lowering and lipid-lowering drugs at 40.35%, 8.25%, respectively (3). Thus, it is crucial to provide more attention to the key barriers of CVD prevention to minimize CVD risk.

Sustainable prevention strategies cannot work effectively without the participation of CVD risk populations. Understanding CVD risk is a prerequisite for adopting a healthy lifestyle and habits conducive to health (6). Risk perception is considered as a critical psychological construct, that affects health behavior change and maintenance (7). A correct risk perception is necessary for an individual to adopt a healthy lifestyle, and risk perception deficiency is considered an additional CVD risk factor (8). Studies have shown that individuals who perceive themselves at a higher risk of CVD are more likely to adopt a healthy lifestyle (9, 10) or demonstrate a willingness to consider future prevention therapy (11). In general, knowledge of the widely promoted risk factors for CVD, such as elderly, smoking, being obese, and high blood pressure, may form the basis of an individual's risk perception (6, 12). Thus, improving knowledge and risk perception is an integral part of behavioral interventions aimed at reducing the incidence of CVD (13). Although there has been considerable research in Western populations, little is currently known about CVD risk perception among Chinese adults.

In regard to CVD risk perception, studies have focused on the measurement, experience, and influence outcomes of risk perception and have emphasized the importance of the accuracy of CVD risk perception (11). Gender, age, education level, socioeconomic status, body mass index (BMI), perceived health status, and other factors have been reported to be associated with CVD risk perception (14–16), while several studies suggest no significant differences for CVD risk perception across age, sex, occupation, and education groups (11, 17). Traditionally, these studies have concentrated on the relationship between variables while ignoring individual differences in CVD risk perception. Although previous studies have applied the single-item risk perception assessment tool, vertical slider scale with numbers, or multi-item measurements, none have reported a cut-off value or classification approach to indicate latent categories of CVD risk perception. Without such categorization, further analysis of the characteristics to distinguish different risk perception categories would be limited. This lack of clarity could affect our ability to identify subgroups of individuals who may be at relatively higher or lower perceived risk for CVD and who may benefit from tailored preventive interventions.

Latent profile analysis (LPA) is a person-centered algorithm that aims to classify individuals into unobserved groupings (latent classes) with similar (more homogeneous) patterns (18). LPA enables us to create and expand theoretical thinking on the existence of various profiles in variables. Applying a person-centered approach to research on CVD risk perception can identify latent profiles that differ from each other in terms of individual perceived CVD risk. In sum, to better understand CVD risk perception and develop targeted risk communication strategies, this study intends to (a) explore CVD risk perception subgroups among community-dwelling adults in South China using LPA, and identify the characteristics of each categories, (b) identify the factors associated with risk misperception compared with objective calculated CVD risk.

## Methods

### Study design and participants

A cross-sectional survey was conducted in Hangzhou, Zhejiang Province, from March to July 2022. Invitations were issued to patients in the endocrinology and physical examination departments of the second affiliated hospital Zhejiang University school of medicine. We recruited individuals who consent to participate after their hospital discharge. The inclusion criteria were (1) being a Zhejiang citizen; (2) age 20–80 years; (3) having no previous diagnosis of CVD; (4) having a full medical examination report within the last 3 months; and (5) being able to read and speak in Mandarin. The participants with critical illness, mental deficiency, pregnancy, or undergoing treatment for a psychiatric disorder were excluded. After the exclusion of responses with incomplete or invalid answers to the questionnaire, a total of 692 participants were included in the final analysis.

### Data collection procedure

Data were collected by well-trained healthcare staff and researchers using the standard protocol and questionnaires with stringent quality control. Before the assessment process, the participants were informed of the topic of the study. A paper-based survey and online survey platform powered by WJX (www.wjx.com) were provided, and participants could choose the one that they preferred. Each participant completed the three parts of the questionnaire, including general information, CVD risk perception assessment, and items for the calculation of 10-year CVD risk. General information and CVD risk perception were self-assessed. If participants were unable to write, the investigators read each item to them, then the questionnaire was completed according to the statements of the participants. The 10-year CVD risk was calculated using an online calculator (https://www.cvdrisk.com.cn/ASCVD/Eval) by the investigator after obtaining indicators from health check report, with the permission of participants. The research was approved by the Ethics Committee of the Second Affiliated Hospital of Zhejiang University School of Medicine (No. 2022-0280).

### Measures

#### General information

Demographic characteristics included age, gender, height (cm), weight (Kg), marital status, education level, ethnic group, employment status, monthly income, smoking and drinking status, family history of CVD, hypertension or diabetes medical history, and subjective health status. BMI was calculated by dividing weight (Kg) by height (m) squared. Family history of CVD referred to participants' having at least one relative (parent or sibling) with myocardial infarction or stroke (19). Smoking and drinking status was determined by the answer to the question, “What is your current smoking/drinking status?” (1 = never smoked/drank, 2 = ever smoked/drank, 3 = currently smoke/drink). Subjective health status was estimated through the answer to the question, “In general, how would you rate your health status?” (1 = very poor, 2 = poor, 3 = fair, 4 = good, 5 = excellent) (19).

#### CVD risk perception

The Attitude and Beliefs about Cardiovascular Disease (ABCD) Risk Questionnaire was employed to measure people's CVD risk perception, which has confirmed validity in a variety of populations (20–22). The original English questionnaire was developed by Woringer et al. (7); we translated and modified the questionnaire into a Chinese version (ABCD-C) in an earlier study (23). The scale contains 26 items and has four dimensions: CVD-related knowledge (8 items), risk perception (8 items), perceived benefits and intention to change physical activity (6 items), and perceived benefits and intention to change dietary habits (4 items). For each item of knowledge, the correct answer was scored as 1, and an incorrect or “I don't know” answer was scored as 0. Values are summed to create a summary score, for which higher values indicate higher CVD-related knowledge. Answer options for the other three dimensions are presented on a 4-point scale and range from 1 = strongly disagree to 4 = strongly agree; a “not applicable” option was added, with a value of 0. Items 15, 21, and 26 were reverse-coded. The ABCD-C was validated and showed good psychometric properties, with a Cronbach's α reliability coefficient of knowledge, risk perception, perceived benefits and intention to change physical activity, and perceived benefits and intention to change dietary habits scale of 0.801, 0.940, 0.900, 0.830, respectively. In the present study, the Cronbach's α for the four dimensions was 0.670, 0.949, 0.885, 0.833, respectively.

#### Objective 10-year CVD risk

The 10-year CVD risk was estimated using the China-PAR (Prediction for Atherosclerotic cardiovascular disease Risk) equation (24), which was developed from the gender-specific Cox proportional hazards model (19). Risk factors in the equation included sex, age, geographic region (Northern China/Southern China), urbanization (urban/rural), treated or untreated systolic blood pressure (mmHg), total cholesterol (mmol/L), high-density lipoprotein cholesterol (HDL-C; mmol/L), current smoking (yes/no), diabetes (yes/no), waist circumference (WC; cm), and family history of CVD (yes/no). Based on the cut-off value of the China-PAR in the Chinese guidelines (25), participants were divided into three categories: low risk (<5%), moderate risk (5%−9.9%) and high risk (≥10%).

#### Statistical analysis

We used LPA to depict the clustering of CVD risk perception using eight dimensions (items) of risk perception as indicators. LPA was performed with the R software package mclust (26) and tidy PLA (27). LPA posits that there is an underlying latent structure that divides a population into mutually exclusive and exhaustive classes (28). We compared model fits on the basis of the Akaike information criterion (AIC), Bayesian information criterion (BIC), sample size-adjusted BIC (aBIC), and the entropy test to determine the number of categories. For these three indices, smaller values indicate a finer balance between model fit and parsimony. High entropy (close to 1) suggests that the bias is minimal for LCA model (29). The bootstrapped likelihood ratio test (BLRT) compares two models with differing numbers of class specifications. A model with fewer latent classes would fit the data better when BLRT does not have a significant test result (*p* > 0.05) (30). The theoretical base for class solutions also was considered in selecting the best number of participant classes (31).

IBM SPSS 26.0 was used to perform descriptive and analytical statistics. Continuous variables were presented as a mean and standard deviation (SD) or interquartile range, while categorical variables were presented as a frequency with the percentage. We used chi-square tests for categorical variables to compare the differences between CVD risk perception groups. The latent classes of CVD risk perception were compared with 10-year CVD risk categories to define correctness of estimation (accurate risk estimation, risk underestimation or overestimation) (32). The mean difference of ABCD-C scores across the latent classes were determined using one-way analysis of variance (ANOVA) and Tukey's multiple comparison test. The effect size partial eta squared (η2) was calculated through the sum of squares of the effect divided by the total sum of the squares; η2 = 0.01 indicates a small effect; η2 = 0.06, medium; and η2 = 0.014, large (33). Multinomial logistic regression models with the full information maximum likelihood method were utilized to determine factors related to the level of CVD risk perception, and correctness of risk estimation. Spearman rank correlations were analyzed between the CVD risk perception latent classes and 10-year CVD risk categories. The correlations of |*r*| = 0.10–0.30, |*r*| = 0.31–0.60, and |*r*| = 0.61–1.00 were considered low, moderate, and high, respectively (34); *p*-values < 0.05 were considered statistically significant.

## Results

### Characteristics of the participants

Table 1 presents the characteristics of the participants. Of the 692 adults, 414 (59.8%) were female, and those aged 20– <40 comprised approximately half of the sample (49.7%). More than half of the participants had an education of bachelor or above (51.6%). Most adults (81.6%) reported good or excellent health status. The 10-year CVD risk, using the China-PAR model, showed that only 87 (12.6%) of participants had high risk, 169 (24.4%) had moderate risk, and 436 (63.0%) had low risk.

**Table 1:** Characteristics of the participants (*N* = 692).

**Characteristic**	***n* (%)**
**Age**	
20– < 40	344 (49.7%)
40– < 60	227 (32.8%)
60–80	121 (17.5%)
**Gender**	
Male	278 (40.2%)
Female	414 (59.8%)
**Educational level**	
Junior school or below	118 (17.0%)
Middle/high school/specialty degree	217 (31.4%)
Bachelor degree or above	357 (51.6%)
**Marital status**	
Single	174 (25.1%)
Married	518 (74.9%)
**Ethnic group**	
Han Chinese	679 (98.1%)
Minority	13 (1.9%)
**Employment status**	
Employed	439 (63.4%)
Unemployed	253 (36.6%)
**Monthly income**	
< 5,000 RMB	314 (45.4%)
≥5,000 RMB	378 (54.6%)
**Smoking status**	
Current smoking	118 (17.1%)
Non-smoking/quit smoking	574 (82.9%)
**Drinking status**	
Current drinking	115 (16.6%)
Non-drinking/quit drinking	577 (83.4%)
**Hypertension**	
Yes	164 (23.7%)
No	528 (76.3%)
**Diabetes**	
Yes	296 (42.8%)
No	396 (57.2%)
**CVD family history**	
Yes	60 (8.7%)
No	632 (91.3%)
**BMI (Kg/m** ^2^ **)**	
< 18.5	63 (9.1%)
18.5–23.9	372 (53.8%)
≥24.0	257 (37.1%)
**Subjective health status**	
Excellent/good	565 (81.6%)
Fair/poor	127 (18.4%)
**10-year CVD risk (%)**	
< 5	436 (63.0%)
5.0–9.9	169 (24.4%)
≥10.0	87 (12.6%)

CVD, cardiovascular disease; BMI, body mass index.

### Latent profile analysis of CVD risk perception

**Figure 1:**
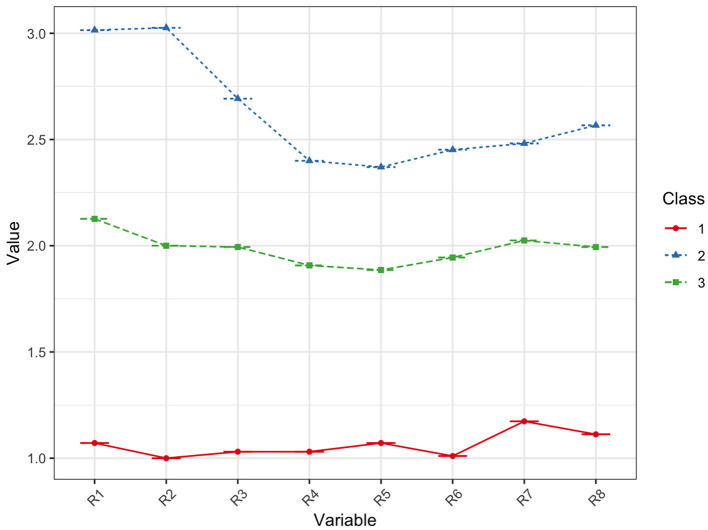
Distribution of three potential classes of CVD risk perception.

Table 2 shows fit indices for latent profile Models 1–4. The AIC, BIC, and aBIC, which were used to test the goodness of the model fit, decreased continuously from Class 1 to Class 4 and declined the fastest for Class 3. The entropy showed an optimal fit for the four models, ranging between 0.990 and 0.998. The two-class model was excluded due to higher AIC, BIC, and aBIC; and the four-class model also was excluded due to having the lowest entropy. As such, the model with three classes was preferred. The mean score of the risk perception was divided into three classes. Figure 1 shows the distribution of the three potential classes with different participant levels.

**Table 2:** Model fit indices for different models.

**Model**	**AIC**	**BIC**	**aBIC**	**BLRT**	**Entropy**	**Category probability**	**Case number**
1-class	11,378.620	11,451.252	11,400.45	–	–	1	692
2-class	8,267.801	8,381.291	8,301.912	0.009	0.988	0.790/0.210	547/145
3-class	5,860.154	6,014.500	5,906.544	0.009	0.998	0.142/0.390/0.468	98/270/324
4-class	4,831.158	5,026.360	4,889.828	0.009	0.990	0.199/0.171/0.383/0.247	138/118/265/171

AIC, Akaike information criterion; BIC, Bayesian information criterion; aBIC, sample size-adjusted BIC; BLRT, bootstrap likelihood ratio test.

### Characteristics of classes

Table 3 presents the scores for CVD risk perception for each group. The other three dimensions of ABCD-C are also shown in the table.

**Table 3:** Four dimensions of ABCD-C for different risk perception groups (*N* = 692).

**Characteristic**	**Low RP class (*n* = 98)**	**Moderate RP class (*n* = 324)**	**High RP class (*n* = 270)**	***F***	***P-*value**	**Partial η^2^**
ABCD-C	43.37 (8.37)^ab^	52.63 (5.76)^ac^	58.69 (4.56)^bc^	261.67	< 0.001	0.43
Knowledge (range = 0–8)	6.27 (1.83)^ab^	5.52 (2.04)^a^	5.73 (1.80)^b^	5.68	0.004	0.02
Risk perception (range = 0–32)	8.50 (0.89)^ab^	15.88 (2.26)^ac^	21.00 (2.26)^bc^	1,310.86	< 0.001	0.79
Perceived benefits and intention to change PA (range = 0–24)	16.78 (4.88)^ab^	18.48 (3.36)^a^	19.05 (2.86)^b^	15.76	< 0.001	0.04
Perceived benefits and intention to change DH (range = 0–16)	11.83 (3.30)^ab^	12.75 (2.09)^a^	12.90 (1.82)^b^	8.85	< 0.001	0.03

ABCD-C, Chinese version of Attitudes and Beliefs about Cardiovascular Disease (ABCD) Risk Perception Questionnaire; PA, physical activity; DH, dietary habits.

The same superscripted letters denote a significant difference between each level of variables in the column; p < 0.05.

Table 4 shows the characteristics of participants in each CVD risk perception group. Except for gender and ethnic group, other characteristics were significantly different among the three classes (*p* < 0.05). Further, a significant positive correlation was found among three latent classes and the 10-year CVD risk (*r*= 0.295, *p* < 0.001).

**Table 4:** Characteristics of individuals among risk perception groups (*N* = 692).

**Characteristic**	**Low RP class (*n* = 98)**	**Moderate RP class (*n* = 324)**	**High RP class (*n* = 270)**	**χ*^2^***	***p*-value**
**Age**					
20– < 40	91 (92.9%)	164 (50.6%)	89 (33.0%)	103.838	< 0.001
40– < 60	4 (4.1%)	102 (31.5%)	121 (44.8%)		
60–80	3 (3.1%)	58 (17.9%)	60 (22.2%)		
**Gender**					
Male	31 (31.6%)	126 (38.9%)	121 (44.8%)	5.617	0.060
Female	67 (68.4%)	198 (61.1%)	149 (55.2%)		
**Educational level**					
Junior school or below	4 (4.1%)	47 (14.5%)	67 (24.8%)	78.168	< 0.001
Middle/high school/ specialty degree	6 (6.1%)	124 (38.3%)	87 (32.2%)		
Bachelor degree or above	88 (89.8%)	153 (47.2%)	116 (43.0%)		
**Marital status**					
Single	51 (52.0%)	94 (29.0%)	29 (10.7%)	70.002	< 0.001
Married	47 (48.0%)	230 (71.0%)	241 (89.3%)		
**Ethnic group**					
Han Chinese	95 (96.9%)	321 (99.1%)	263 (97.4%)	3.086	0.214
Minority	3 (3.1%)	3 (0.9%)	7 (2.6%)		
**Employment status**					
Employed	91 (92.9%)	214 (66.0%)	134 (49.6%)	59.718	< 0.001
Unemployed	7 (7.1%)	110 (34.0%)	136 (50.4%)		
**Monthly income**					
< 5,000 RMB	23 (23.5%)	139 (42.9%)	152 (56.3%)	32.765	< 0.001
≥5,000 RMB	75 (76.5%)	185 (57.1%)	118 (43.7%)		
**Smoking status**					
Current smoking	1 (1.0%)	56 (17.3%)	61 (22.6%)	23.680	< 0.001
Non-smoking/quit smoking	97 (99.0%)	268 (82.7%)	209 (77.4%)		
**Drinking status**					
Current drinking	6 (6.1%)	62 (19.1%)	47 (17.4%)	9.394	0.009
Non-drinking/quit drinking	92 (93.9%)	262 (80.9%)	223 (82.6%)		
**Hypertension**					
Yes	3 (3.1%)	72 (22.2%)	89 (33.0%)	36.288	< 0.001
No	95 (96.9%)	252 (77.8%)	181 (67.0%)		
**Diabetes**					
Yes	3 (3.1%)	138 (42.6%)	155 (57.4%)	86.765	< 0.001
No	95 (96.9%)	186 (57.4%)	115 (42.6%)		
**CVD family history**					
Yes	10 (10.2%)	18 (5.6%)	32 (11.9%)	7.712	0.021
No	88 (89.8%)	306 (94.4%)	238 (88.1%)		
**BMI (Kg/m** ^2^ **)**					
< 18.5	10 (10.2%)	30 (9.3%)	23 (8.5%)	20.172	< 0.001
18.5–23.9	70 (71.4%)	173 (53.4%)	129 (47.8%)		
≥24.0	18 (18.4%)	121 (37.3%)	118 (43.7%)		
**Subjective health status**					
Excellent/good	92 (93.9%)	266 (82.1%)	207 (76.7%)	14.297	0.001
Fair/poor	6 (6.1%)	58 (17.9%)	63 (23.3%)		
**10-year CVD risk (%)**					
< 5	95 (97.0%)	211 (65.1%)	130 (48.1%)	76.328	< 0.001
5.0–9.9	2 (2.0%)	70 (21.6%)	97 (35.9%)		
≥10.0	1 (1.0%)	43 (13.3%)	43 (15.9%)		

CVD, cardiovascular disease; BMI, body mass index.

Class 1, “low risk perception,” comprised 14.2% (98/692) of the sample. This class had a low risk perception score (8.50 ± 0.89). Moreover, the participants in this group were younger, had higher education and better CVD related knowledge, reported better health status, and had a lower 10-year CVD risk than those in other groups. In addition, participants with low risk perception had a lower perceived benefit of physical activity and healthy dietary habits and, thus, less intent to change behavior.

Class 2, “high risk perception,” represented 39.0% (270/692) of the sample. The risk perception score of this class was the highest, near twice that of Class 3 and three times that of Class 1, indicating that Class 2 had the highest level of CVD risk perception among the three classes. Most adults in Class 2 had a lower education level and monthly income but a higher BMI and 10-year CVD risk.

Class 3, “moderate risk perception,” comprised the highest proportion of the sample, at 46.8% (324/692). This class showed better risk perception than the other two classes. The perceived benefits and intention to change physical activity or dietary habit was higher in this class than those in Class 1.

### Multinomial logistics regression

Table 5 shows the multinational logistic regression results. Compared with low-risk perception class, participants aged 40–60 (*OR* = 4.52, 95% *CI* = 1.24–16.45), monthly income more than 5000 RMB (*OR* = 1.45, 95% *CI* = 1.02–2.05), diabetes (*OR* = 4.81, 95% *CI* = 1.07–21.74), and perceived benefits and intention to change PA (*OR* = 1.10, 95% *CI* = 1.01–1.20) were more likely to be in moderate risk perception class. Participants aged 40–60 (*OR* = 6.94,95% *CI* = 1.86–25.84), married (*OR* = 4.52, 95% *CI* = 2.30–8.90), diabetes (*OR* = 6.26, 95% *CI* = 1.34–29.17), good or excellent health status (*OR* = 3.23, 95% *CI* = 1.15–9.10), and perceived benefits and intention to change PA (*OR* = 1.16, 95% *CI* = 1.05–1.27) were more likely to be in high risk perception class than low risk perception class.

**Table 5:** Multinomial logistic regression on CVD risk perception.

**Characteristic**	**Moderate RP class**		**High RP class**	
	**OR**	**95% CI**	**OR**	**95% CI**
**Age (Ref. 20–**<**40)**				
40– < 60	4.52^*^	1.24–16.45	6.94^*^	1.86–25.84
60–80	1.80	0.20–16.36	1.79	0.19–16.99
**Educational level (Ref. Junior school or below)**				
Middle/high school/specialty degree	3.01	0.56–16.37	1.33	0.24–7.38
Bachelor degree or above	1.25	0.19–8.07	4.13	0.59–28.78
**Marital status (Ref. Single)**				
Married	1.08	0.61–1.89	4.52^*^	2.30–8.90
**Employment status (Ref. Unemployed)**				
Employed	0.71	0.16–3.08	1.83	0.40–8.28
**Monthly income (Ref**. < **5,000 RMB)**				
≥5,000 RMB	1.45^*^	1.02–2.05	1.20	0.81–1.77
**Smoking status (Ref. No)**				
Current smoking	1.61	0.14–18.47	2.43	0.26–23.10
**Drinking status (Ref. No)**				
Current drinking	2.82	0.79–10.11	2.57	0.91–7.23
**Hypertension (Ref. No)**				
Yes	1.78	0.39–8.22	3.05	0.66–14.18
**Diabetes (Ref. No)**				
Yes	4.81^*^	1.07–21.74	6.26^*^	1.34–29.17
**CVD family history (Ref. No)**				
Yes	0.64	0.26–1.59	2.08	0.81–5.29
**BMI (Ref. 18.5–23.9)**				
< 18.5	1.76	0.78–3.97	2.39	0.97–5.85
≥24.0	0.94	0.48–1.87	1.31	0.65–2.66
**Subjective health status (Ref. Fair/poor)**				
Excellent/good	2.09	0.76–5.72	3.23^*^	1.15–9.10
**10–year CVD risk (**<**5%)**				
5.0%−9.9%	1.64	0.21–12.61	2.30	0.29–18.36
≥10.0%	2.81	0.14–56.94	1.94	0.09–40.29
Knowledge (unit: one score)	0.88	0.75–1.03	0.98	0.83–1.16
Perceived benefits and intention to change PA (unit: one score)	1.10^*^	1.01–1.20	1.16^*^	1.05–1.27
Perceived benefits and intention to change DH (unit: one score)	0.99	0.88–1.12	0.95	0.83–1.09

CVD, cardiovascular disease; BMI, body mass index; PA, physical activity; DH, dietary habits; RP, risk perception.

* p < 0.05.

### Perceived vs. calculated CVD risk

Compared to absolute 10-year CVD risk based on China-PAR, a third of participants (30.1%) correctly estimated their CVD risk, 63.3% overestimated it and 6.6% underestimated it (Table 6). More than half individuals (50.6%) with high risk underestimated their CVD risk.

**Table 6:** Meshing table between perceived CVD risk and calculated 10-year CVD risk.

**Perceived CVD risk**	**China-PAR risk score**			**Total**
	**Low**	**Moderate**	**High**	
Low	95	2	1	98 (14.2)
Moderate	211	70	43	324 (46.8)
High	130	97	43	270 (39.0)
Total	436 (63.0)	169 (24.4)	87 (12.6)	692 (100)

CVD, cardiovascular disease; China-PAR, Prediction for Atherosclerotic cardiovascular disease Risk; Numbers in absolute, the percentage of total was calculated in column.

**Figure 2:**
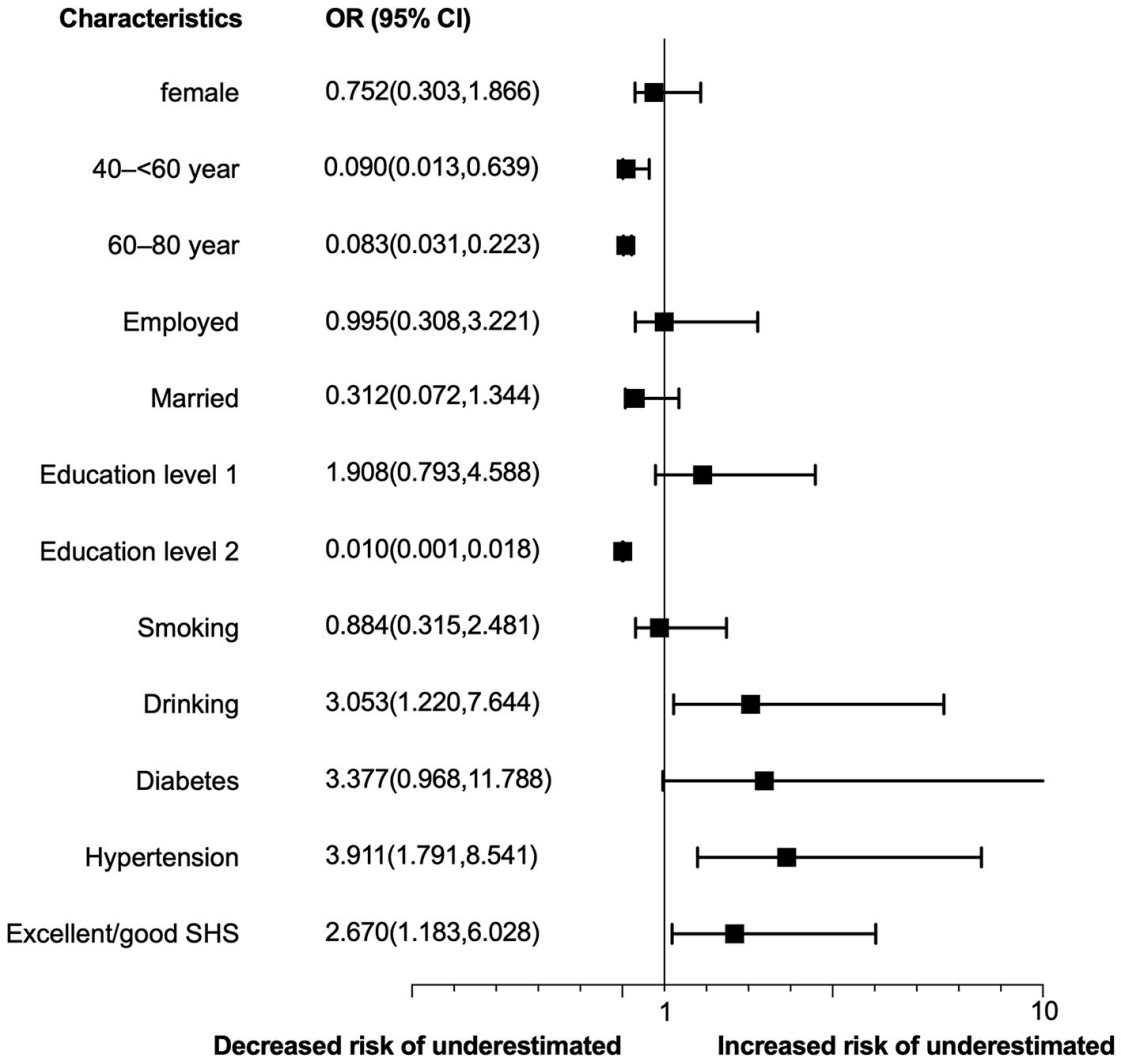
Determinants of CVD risk underestimation. Education level 1 refers to middle/high school/specialty degree; education level 2 refers to bachelor degree or above; SHS, subjective health status.

We combined individuals who accurately estimate or overestimate their CVD risk together to explore the risk factors associated with risk underestimation, recognizing that underestimation has more detrimental impact on CVD prevention than accurate estimation or overestimation. In the multivariable-adjusted analysis, underestimation of CVD risk was associated with hypertension (*OR* = 3.91, 95% *CI* = 1.79–8.54), drinking (*OR* = 3.05, 95% *CI* = 1.22–7.64), better subjective health status (*OR* = 2.67, 95% *CI* = 1.18–6.03) (Figure 2).

## Discussion

The current study is considered the first work that uses the LPA approach to explore subgroups of CVD risk perception among community adults in South China. Our findings determined three latent classes based on the risk perception score of the ABCD-C questionnaire. Close to half of the participants (46.8%) were classified as having moderate perceived CVD risk, while those who were assessed as having low risk perception accounted for the smallest proportion (14.2%). Advanced age, higher monthly income, diabetes, better health status, and having higher perceived benefits and intention to change PA were significantly related to higher perceived CVD risk. Compared to absolute 10-year CVD risk based on China-PAR, only a third of participants (30.1%) correctly estimated their CVD risk. Individuals with hypertension, drinking and better subjective health status were associated with CVD risk underestimation.

Regarding CVD risk perception measurement, a large number of studies have focused on a single-item risk perception assessment tool application, with the choices of “low,” “intermediate,” and “high” risk (35) or a scale of a 0–100% (36) chance to get CVD within the next 10 years. Importantly, because a person's interpretation of a qualitative risk expression (e.g., high vs. low) can vary, any qualitative statements should be accompanied by a numerical description (37). A lower level of numeracy skill was associated with an overestimation of risk when using a numerical measurement (38, 39). These results suggest that a CVD risk perception level cannot be classified correctly by a single item. In addition, a general reference range for what was considered high or low risk on a scale was absent. The ABCD Risk Questionnaire was developed to measure CVD knowledge and risk perception (7) and has confirmed validity in English (7), Hungarian (20), Dutch (21), and Malay (22). The risk perception dimensions of the ABCD are represented by eight items that describe the perceived susceptibility and severity of individuals for CVD (7). Thus, we use these eight items as a screening tool to classify people through LPA analysis. Our findings demonstrated the distribution of CVD risk perception in Chinese adults, which provided valuable insight into the current status of CVD risk perception.

Further, more than half of the participants were classified into the moderate CVD risk perception group in this study, and 39.0% were in the high-risk perception class. Considering that risk perception is recognized as a modifiable determinant of health-related behaviors (40, 41), it is vital to identify subgroups on which to focus to maximize the maintenance of risk perception or improve the level of CVD risk perception through targeted intervention to prevent a decline in the level of risk perception. Our study highlighted that individuals who were older, had a higher monthly income, and had diabetes were more prone to perceive higher CVD risk, which has been confirmed in earlier studies (32, 38, 42). Our study also found that people who perceived better health status were more likely to be in a high-risk perception class, which was inconsistent with the findings in Korean blue-collar workers (15). There may be an interaction between risk perception and personal health status. According to the health belief model (43), people who perceived themselves as at higher health risk tend to pay more attention to their health status and to improve their health through behavioral changes. In turn, perceived health status is critical for capturing health risk information. Moreover, due to the cross-sectional design of our study, we could not establish a causal relationship between risk perception and subjective health status. Nonetheless, our findings also highlight the necessity of better health status maintenance in the improvement of CVD risk perception.

Although the majority of the participants (63.0%) in our study were at low 10-year CVD risk based on China-PAR, only 14.2% fell into the low-risk perception class through LPA. This presents a unique opportunity to investigate the potential causes. The time horizon used for risk estimation would influence individuals risk perception (44). Specifically, respondents were more likely to consider their risk “high to very high” when shown the lifetime CVD risk, than when presented the 10-year CVD risk. The items of risk perception assessment used in this study include both short time (10-year, item 12–13) and lifetime risk (item 9–11) to reflect the individuals' risk perception comprehensively. This maybe the critical reason accounting for a lower risk perception proportion in our analysis. Importantly, because a person's perception of risk expression (format, time horizon) can vary, any qualitative statements should be accompanied by the condition description of such a statement.

The accuracy of perceived CVD risk was assessed by comparing participants' risk perception responses to their objective calculated risk derived by the Framingham adult treatment panel (ATP) III model (45), Pooled cohort equations (PCE) (46) or the China-PAR (19). The China-PAR was developed and validated to predict 10-year CVD risk, using data from four contemporary Chinese cohorts. A significant positive correlation was found between three latent classes and 10-year CVD risk calculated by the China-PAR (*r* = 0.295, *p* < 0.001), but only 30.1% of participants estimated their CVD risk correctly. Interestingly, more participants (63.3%) overestimated their CVD risk than underestimated (6.6%) it in our study. These findings were consistent with previous studies (risk overestimation: 39%−72.2%) (11, 35). While, a number of studies reported that participants generally have optimistic bias and underestimate their risk of having heart attack or stroke in the future when compared to age and sex matched individuals (40). These findings supported the importance of marked reference and cut-off point, when reporting accurate levels of CVD risk perception. Nonetheless, risk underestimation (too optimistic) had more detrimental influence on health behaviors toward CVD prevention, such as the adherence of medication (40), and lifestyle modifications (47, 48), so as to increase the absolute risk of CVD event. We also revealed that risk underestimation was particularly prevalent among individuals with high CVD risk. Efforts should be made to increase the awareness and accurate perception toward CVD risk in these population, in order to promote proactive health behaviors and self-management of CVD.

To our surprise, hypertension was a determinant of CVD risk underestimation, even though those individuals have had regular medical interaction and lifestyle education (49). Figure 2 showed that diabetes also was a potential factor for risk underestimation. Effective risk communication is essential for promoting public awareness and understanding of potential hazards, as well as empowering individuals and communities to make informed decisions and take appropriate actions to reduce their exposure and vulnerability (50). However, many institutions and organizations have not yet fully integrated risk communication into their policies and practices. As a result, there may be gaps in the dissemination of accurate, timely, and relevant information about CVD risks and their impacts, as well as in the engagement and participation of diverse stakeholders in risk management and decision-making processes. Therefore, it is important to prioritize and support the development of risk communication strategies that are inclusive, adaptive to the contexts of individuals' characteristics, and that integrate it into routine healthcare visits to improve the understanding of CVD risk.

This study has several limitations. First, the study subjects were recruited through convenience sampling from a single tertiary hospital. Although this hospital is a prominent regional central hospital in south China, catering patients from a wide geographic aeras, and serving as a designated site for physical examination for various companies, these population who participate in our investigation were more likely to be younger, higher education level and income. In addition, we also recruited subjects in the endocrinology department, resulting in a higher proportion of participants with diabetes (42.8%) or hypertension (23.7%). Consequently, the generalizability of the study findings may be limited, in particular, the risk perception category cutoff values maybe inappropriate to healthier adults with lower education levels and elderly individuals. Second, we evaluated risk perception based on comprehensive tool covering 10-year risk and lifetime risk, whereas objective CVD risk calculation only followed by 10-year risk. Thus, our conclusions were drawn based on a comparison with 10-year CVD risk. Third, the study was cross-sectional descriptive, limiting our ability to generalize the results related to the interaction between individual risk factors and risk perception. Similarly, we do not explore how individuals form their perception of CVD risk. These limitations highlight the need for future research to address these issues to gain a more comprehensive understanding of CVD risk perception and its impact on preventive behaviors and health outcomes.

## Conclusions

As an overview of subgroups of CVD risk perception based on a person-centered approach, this study identifies three classes of CVD risk perception among adults in south China. The majority of participants have a moderate level of CVD risk perception. Advanced age, higher monthly income, diabetes and better health status were significantly related to higher perceived CVD risk. Compared to absolute 10-year CVD risk, only a third of participants correctly estimated their risk. Notably, individuals with hypertension, drinking and better subjective health status were associated with CVD risk underestimation. Healthcare professionals should pay attention to the indicators for different categories and identify underestimation group as early as possible. To improve the accuracy of CVD risk perception, healthcare professionals should focus on effective risk communication among these population.

## Data availability statement

The raw data supporting the conclusions of this article will be made available by the authors, without undue reservation.

## Ethics statement

The studies involving human participants were reviewed and approved by the Institutional Review Board of the Second Affiliated Hospital of Zhejiang University School of Medicine (No. ID: 2022-0280). The patients/participants provided their written informed consent to participate in this study.

## Author contributions

ZG: conceptualization, investigation, statistical analyses, and writing of the paper. YY: data curation. YF, CD, and EG: investigation and data curation. NC, YZ, QY, and JJ: methodology, paper review, and editing. JJ: supervision. All authors contributed to the article and approved the submitted version.
